# Targeting CDK11 in Rhabdoid Tumor of the Kidney

**DOI:** 10.3390/cancers18020261

**Published:** 2026-01-14

**Authors:** Yuki Murakami, Kamhung Lam, Shinsuke Fukui, Elizabeth Helmke, Kenneth A. Iczkowski, Yueju Li, Noriko Satake

**Affiliations:** 1 Department of Pediatrics, School of Medicine, University of California, Davis, Sacramento, CA 95817, USA; yuuki1228@asahikawa-med.ac.jp (Y.M.); khvlam@health.ucdavis.edu (K.L.); shfukui@health.ucdavis.edu (S.F.); ewhelmke@ucdavis.edu (E.H.); 2 Comprehensive Cancer Center, University of California, Davis, Sacramento, CA 95817, USA; 3 Department of Pathology and Laboratory Medicine, School of Medicine, University of California, Davis, Sacramento, CA 95817, USA; kaiczkowski@health.ucdavis.edu; 4 Department of Public Health Sciences, School of Medicine, University of California, Davis, Sacramento, CA 95817, USA; yjlli@health.ucdavis.edu

**Keywords:** rhabdoid tumor, CDK11 inhibitor, cell cycle, apoptosis, RNA splicing

## Abstract

Background: Rhabdoid tumor of the kidney (RTK) is a highly aggressive pediatric malignancy characterized by biallelic *SMARCB1* loss, resulting in aberrant MYC pathway activation and cell cycle regulation. MYC-activated tumors are vulnerable in splicing functions and sensitive to splicing inhibitors. Therefore, in this study, cyclin-dependent kinase 11 (CDK11), which regulates both cell cycle and RNA splicing, was tested as a therapeutic target in RTK. Methods: *CDK11A/B* expression was analyzed using the TARGET-RT database. The therapeutic efficacy of the CDK11 inhibitor OTS964 was evaluated in two RTK cell lines (G401 and JMU-RTK-2) and a JMU-RTK-2 xenograft mouse model. Cytotoxicity, apoptosis, cell cycle, and RNA splicing were examined using the Sulforhodamine B assay, immunoblotting, flow cytometry, and RT-PCR. Results: *CDK11B*, but not *CDK11A*, was significantly upregulated in RTK and correlated with the poor survival. OTS964 inhibited RTK cell growth in vitro with the IC50 of 33.1 nM (G401) and 19.3 nM (JMU-RTK-2) and significantly prolonged survival in vivo (median survival: 46.5 vs. 37.0 days, *p* < 0.01) without marked toxicity. Mechanistically, OTS964 induced G2/M cell cycle arrest and p53 upregulation, disrupted RNA splicing via SF3B1 dephosphorylation, and ultimately led to apoptosis through caspase-3 activation. Conclusions: CDK11 inhibition by OTS964 effectively suppresses RTK growth through cell cycle arrest and RNA splicing inhibition, leading to apoptosis. OTS964 shows potent anti-tumor activity and tolerability, supporting CDK11 as a promising therapeutic target for RTK and related *SMARCB1*-deficient cancers.

## Simple Summary

Rhabdoid tumor of the kidney (RTK) is a rare and aggressive childhood cancer with very poor survival. Current treatments are limited, and new therapies are urgently needed. CDK11 controls both cell cycle and RNA splicing. CDK11 was highly expressed in RTK and associated with poor patient outcomes. A CDK11 inhibitor OTS964 inhibited tumor cell growth in RTK cells and our mouse model. OTS964 induced cell cycle arrest and disrupted RNA splicing, leading to cell death. Importantly, in our mouse model, OTS964, as a single drug treatment, even at low doses, showed significant efficacy, with no major side effects. These results suggest that blocking CDK11 could become a promising new treatment strategy for RTK and similar cancers with SMARCB1 loss.

## 1. Introduction

Rhabdoid tumor (RT) is a rare, highly malignant tumor arising in the brain as atypical teratoid/RT (AT/RT), in the kidney as RT of the kidney (RTK), or in the soft tissues as malignant RT (MRT). RT incidence peaks between 1 and 4 years old [1]. RT has extremely poor prognosis despite multimodal treatment, including surgery, chemotherapy, and radiation, with a median survival time of less than 3 years for AT/RT and less than 1 year for RTK and MRT [1,2]. These poor outcomes result from intrinsic chemoresistance, rapid tumor growth, and the absence of effective targeted therapies [3,4]. Therefore, novel therapeutic strategies are urgently needed.

RT is characterized by biallelic *SMARCB1* loss, leading to aberrant MYC pathway activation and cell cycle disruption at both the G1/S and G2/M transitions [5,6,7]. MYC pathway activation shows vulnerabilities in splicing functions and increases sensitivity to splicing inhibitors [8,9,10]. Therefore, targeting RNA splicing and the cell cycle is a reasonable therapeutic strategy.

Recent studies highlight CDK11 as a cyclin-dependent kinase regulating both cell cycle and RNA splicing [10,11]. CDK11 promotes phosphorylation of splicing factors such as SF3B1, thereby regulating RNA splicing [10,12,13]. In addition, CDK11 regulates G2/M progression and promotes cell proliferation, while its inhibition induces apoptosis through caspase-3 activation [14,15]. Given the MYC-activated characteristic of RT and its dual vulnerability to splicing inhibition and cell cycle dysregulation, we hypothesized that CDK11 is a promising therapeutic target in RT.

Humans possess two highly homologous paralogs, *CDK11A* and *CDK11B* [10,13,14]. They encode proteins with 99% sequence identity and largely overlapping functions; therefore, they are often not distinguished in studies [10,13]. Previous studies have reported that *CDK11A* and *CDK11B* expression patterns may vary across cancers. For instance, both *CDK11A* and *CDK11B* are overexpressed in esophageal cancer [16], while only *CDK11B* is upregulated in breast cancer [17]. Notably, the expression status and clinical relevance of CDK11 in RT, including RTK, have not been well characterized. In this study, our analysis of publicly available RTK datasets revealed that CDK11, particularly *CDK11B*, is highly expressed and is associated with poor clinical outcomes, providing a strong rationale for targeting CDK11 in RTK.

OTS964 is a small-molecule CDK11 inhibitor that directly binds to CDK11 and has demonstrated therapeutic efficacy in multiple cancer models [18,19,20]. However, OTS964 has never been evaluated in RT, and its therapeutic potential in *SMARCB1*-deficient cancers remains unknown.

In this study, using RTK, which has one of the poorest prognoses among RT subtypes, we evaluated the therapeutic potential of OTS964 both in vitro and in vivo. We also investigated the mechanisms by which OTS964 induced apoptosis, cell cycle arrest, and inhibition of RNA splicing via the SF3B1 pathway in RTK.

## 2. Materials and Methods

### 2.1. Reagents

OTS964 (MedChemExpress; Monmouth Junction, NJ, USA; HY-12467) was reconstituted in dimethyl sulfoxide (DMSO, 100 µM) (Mylan; Canonsburg, PA, USA; 6745717850) and stored at −80 °C. For all the in vitro experiments, OTS964 was diluted in culture media so that the final DMSO concentration did not exceed 0.1%.

### 2.2. Cell Lines

Human RTK cell line G401 was provided by the Memorial Sloan Kettering Cancer Center (New York, NY, USA) and JMU-RTK-2 was purchased from the Japanese Collection of Research Bioresources (JCRB; Osaka, Japan) cell bank. G401 and JMU-RTK-2 cells were maintained at 37 °C in a 5% CO_2_ incubator. G401 cells were cultured in McCoy’s 5A (Thermo Fisher Scientific; Waltham, MA, USA; 16600082) supplemented with 10% heat-inactivated fetal bovine serum (Corning; Corning, NY, USA; 35-015-CV) and 100 U/mL penicillin and 100 μg/mL streptomycin (Thermo Fisher Scientific; 15140122). JMU-RTK-2 cells were cultured in DMEM (Thermo Fisher Scientific; 11965092) supplemented with 10% heat-inactivated fetal bovine serum and 100 U/mL penicillin and 100 μg/mL streptomycin. Cells were used within the first 30 passages.

### 2.3. Sulforhodamine B (SRB) Assay

SRB assays were performed as previously described in a published protocol [21]. Cells were plated in triplicate onto 96-well tissue culture plates in 7500 cells/well for G401 and JMU-RTK-2. Cells were incubated for 24 h and then exposed to vehicle control (DMSO) or OTS964 for 48 h. Cells were fixed with trichloroacetic acid (final concentration 10%) at 4 °C for more than 1 h, washed, then dried at room temperature, stained with 0.057% SRB in 1% acetic acid, and dye was then solubilized with 10 mM Tris base. The absorbance was measured at 490 nm using a microplate reader (Molecular Devices; San Jose, CA, USA). The absolute optical densities were normalized to time-matched control cells and expressed as percent viabilities.

### 2.4. In Vivo Experiment

JMU-RTK-2 cells (1 × 10^6^) mixed with Matrigel (BD Bioscience; Milpitas, CA, USA; 354230) and DMEM at a 1:1 ratio were subcutaneously injected into the flank of 4-week-old female NU/J athymic nude mice (Jackson; Bar Harbor, ME, USA) using our approved Institutional Animal Care and Use Committee (IACUC) protocol (Approval No. 23528, approved on 23 August 2023). Once tumors reached 80 mm^3^, mice were randomly assigned to treatment groups and treated daily by oral gavage for 14 days with vehicle (10% DMSO/90% corn oil) or 25 mg/kg of OTS964. Tumor volumes were measured every other day using digital calipers and calculated using the following formula: tumor volume = (long diameter × short diameter^2^)/2. Mice were monitored daily and euthanized when the tumor volumes were over 2000 mm^3^ or the long axes of the tumors were over 2 cm in accordance with the IACUC policy on humane endpoints.

### 2.5. Hematoxylin and Eosin (H&E) Staining

Tumors were fixed in 10% formalin, embedded in paraffin, and sectioned (5 μm). The sections were deparaffinized, rehydrated, and stained with H&E using standard protocols.

### 2.6. Immunoblot Analysis

Immunoblotting was performed as previously described [22]. Total proteins were extracted with Pierce™ RIPA Lysis and Extraction Buffer (Thermo Fisher Scientific; 89900) with cOmplete^TM^ Protease Inhibitor tablets (Roche; Basel, Switzerland; 04693116001). A total of 5–15 µg of protein was loaded on a 10–12% Tris-Glycine SDS-PAGE gel then transferred to a PVDF membrane. Blots were blocked with 1% BSA in PBS-T for 1 h at room temperature, incubated with primary antibodies overnight at 4 °C, and then incubated with secondary antibodies for 1 h at room temperature. Primary antibodies and incubation conditions were 1:1000 for mouse anti-p53 (Santa Cruz Biotechnology, Inc.; Dallas, TX, USA; sc-126), Actin (Santa Cruz Bio; sc-8432), and rabbit anti-cleaved caspase-3 (Cell Signaling; Danvers, MA, USA; #9661), PARP (Cell Signaling; #5625), phosphorylation of SF3B1 (Cell Signaling; #25009) and CDK11 (Cell Signaling; #5524) antibodies, and 1:200 for mouse anti-SF3B1 antibody (Santa Cruz Bio; sc-514655), cyclin B1 (Santa Cruz Bio; sc-245), and p21 (Santa Cruz Bio; sc-6246). Secondary antibodies and incubation conditions were 1:10,000 for goat anti-mouse horseradish peroxidase (HRP) (Abcam; Waltham, MA, USA; ab97023) and 1:2000 for goat anti-rabbit HRP antibodies (Cell Signaling; #7074). Blots were washed, then incubated with SuperSignal^TM^ West Femto Maximum Sensitivity Substrate (Thermo Fisher Scientific; 34095) for imaging. Bands were visualized using the ChemiDoc MP Imaging System (Bio-Rad; Hercules, CA, USA). Bands from the blot depicted were digitally quantified and the chemiluminescence intensities were normalized to actin.

### 2.7. Cell Cycle Synchronization

Cells were plated at a density of 1.2 × 10^5^ cells/mL in 2 mL of media in 6-well plates and incubated for 24 h at 37 °C with 5% CO_2_. The first block was initiated by replacing the media with fresh media containing 2 mM thymidine and incubating for 17 h. Cells were then released into fresh thymidine-free media for 7 h. A second block was applied using 2 mM thymidine-containing media. For drug treatment, thymidine-containing media was replaced with media containing DMSO or OTS964, and cells were treated for 8 h for G401 and 12 h for JMU-RTK-2.

### 2.8. Cell Cycle Assay

After the double-thymidine block and OTS964 treatment, the cells were then trypsinized, washed twice with PBS, and fixed overnight at 4 °C in 0.5 mL of PBS and 1 mL of absolute ethanol (≥99.5%). The fixed cells were incubated with 25 U/mL RNase (Thermo Fisher Scientific; R1253) at room temperature for 20 min, and propidium iodide (PI) solution (Thermo Fisher Scientific; P3566) was added at a final concentration of 50 µg/mL. The cells were incubated with PI solution at room temperature for at least 30 min. The cell cycle was assessed by the BD LSRII flow cytometer (Becton, Dickinson and Company; Franklin Lakes, NJ, USA). For each sample, 50,000 events were consistently acquired. Cell cycle profiles were analyzed as the percentage of cells in the G0/G1, S, and G2/M phases relative to the total acquired events. This percentage-based normalization ensures that phase distribution is directly comparable between groups.

### 2.9. Reverse-Transcription-PCR (RT-PCR)

Total RNA was extracted and purified with the PureLink™ RNA Mini Kit (Thermo Fisher Scientific; 12183025). Total RNA (100 ng per reaction) was used as input for cDNA synthesis using the High-Capacity cDNA Reverse Transcription Kit (Thermo Fisher Scientific; 4368814), following the manufacturer’s protocol. Following reverse transcription, PCR was performed with the following primer pairs: 5′-GAACCAAAATCACTTTCCCCAAGGAAGG-3′ and 5′-AATGAGGTCCCCACGTTTCTCGGGTGT-3′ for *DNAJB1* (Thermo Fisher Scientific; 10336022). PCR cycling conditions were: 94 °C for 3 min; then 30 cycles of 94 °C for 30 s, 58 °C for 90 s, and 72 °C for 90 s; followed by a final extension at 72 °C for 5 min. The products were analyzed by electrophoresis on a 2% agarose gel containing GelRed^®^ Nucleic Acid Gel Stain (Biotium; Fremont, CA, USA; 41003). Bands were visualized by Gel Doc EZ Imager version 6.1 (Bio-Rad).

### 2.10. Statistics

Statistical analyses were performed using GraphPad Prism software package 10.4 (GraphPad; San Diego, CA, USA). All in vitro experiments were independently repeated at least three times. Unpaired *t*-tests were employed to compare the two groups. Statistical significance for survival time was determined by the log-rank test. Kaplan–Meier survival curves were created. Statistical significance for tumor volume curves was determined by a mixed-effects model (REML) with repeated measures. All reported *p*-values of <0.05 were considered statistically significant. All raw data used for statistical analyses are available in Supplementary File S1.

## 3. Results

### 3.1. CDK11 Is Highly Expressed in RTK and Correlates with a Poor Prognosis

**Figure 1:**
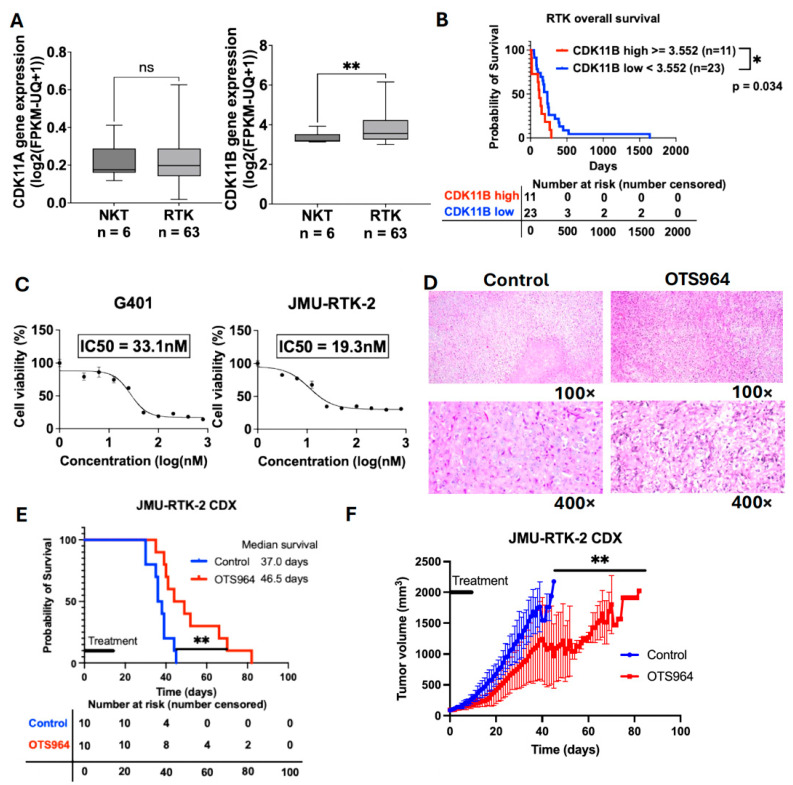
Analysis of CDK11 expression and the therapeutic effects of the CDK11 inhibitor in RTK. (**A**) *CDK11A* and *CDK11B* expression levels in normal kidney tissues (NKT, n = 6) and RTK tissues (n = 63) obtained from the Therapeutically Applicable Research To Generate Effective Treatments (TARGET)-RT database (** *p* < 0.01). (**B**) Overall survival of RTK patients stratified by *CDK11B* expression (Median survival *CDK11B* high: 120 days, *CDK11B* low: 232 days, * *p* < 0.05) [Data from the TARGET-RT database]. (**C**) Dose–response curves and IC50 values of OTS964 in G401 (**left**) and JMU-RTK-2 (**right**) cells by determined by SRB assay (n = 3). (**D**) H&E staining of tumors from JMU-RTK-2 xenografts mice (100× and 400× magnification). (**E**) Kaplan–Meier survival curves of JMU-RTK-2 xenograft mice treated with vehicle or OTS964 (median survival: 37 days vs. 46.5 days, respectively, n = 10 per group, ** *p* < 0.01). (**F**) Tumor volume measurements of JMU-RTK-2 xenograft mice treated with vehicle or OTS964 (n = 10 per group, ** *p* < 0.01). Data are presented as the mean ± SD.

Based on the Therapeutically Applicable Research To Generate Effective Treatments (TARGET)-RT database accessed via the UCSC Xena platform [23], *CDK11B*, but not *CDK11A*, expression was significantly higher in RTK tissues (n = 63) compared to normal kidney tissues (n = 6) (Figure 1A, *p* = 0.008). Furthermore, patients who have RTK with high *CDK11B* expression (n = 11) had significantly shorter overall survival than those with low expression (n = 23) (median survival: 120 vs. 232 days, *p* = 0.035) (Figure 1B), suggesting that CDK11 is a potential therapeutic target in RTK. In addition, we compared *CDK11B* expression levels of RTK, with other pediatric renal tumors, including Wilms tumor (WT), and clear cell sarcoma of the kidney (CCSK), using TARGET-PANCAN data obtained from the UCSC Xena platform [23]. *CDK11B* expression in RTK was comparable to that in WT and significantly higher than that in CCSK (Supplementary Figure S1A). Notably, high *CDK11B* expression was not associated with overall survival in WT and CCSK (Supplementary Figure S1B); however, the survival analysis in CCSK should be interpreted with caution due to the limited sample size. These findings indicate that the prognostic significance of *CDK11B* expression appears to be specific to RTK.

### 3.2. A CDK11 Inhibitor OTS964 Is Cytotoxic in Two RTK Cell Lines and Therapeutic in an In Vivo RTK Mouse Model

OTS964 inhibits the protein products of both *CDK11A* and *CDK11B* [10,13]. Based on the SRB assay, OTS964 significantly inhibited the cell growth in G401 and JMU-RTK-2 cell lines in a dose-dependent manner with the calculated IC50 values of 33.1 nM (G401) and 19.3 nM (JMU-RTK-2) (Figure 1C). Next, we examined the in vivo therapeutic efficacy of OTS964 using a mouse xenograft model. We employed JMU-RTK-2, established from metastatic cerebrospinal fluid lesions, for our in vivo study to recapitulate the clinical characteristics of advanced RTK [24]. Mice engrafted with JMU-RTK-2 tumors were treated with 25 mg/kg of OTS964 or vehicle control. Based on previous studies showing severe bone marrow suppression at higher doses (40–100 mg/kg) [25,26], we selected 25 mg/kg of OTS964 to minimize dose-limiting toxicity while maintaining therapeutic potential in our RTK model. A total of 10 mice were included in each group, all of which developed tumors, and tumors from all mice were collected at the study endpoint. We confirmed the development of the RTK tumors in the control and the OTS964 groups by H&E staining of one representative mouse per group (Figure 1D). OTS964 significantly prolonged overall survival (median survival: 46.5 days vs. 37.0 days) (Figure 1E) and significantly suppressed tumor progression (*p* = 0.002) (Figure 1F), supporting CDK11 as a therapeutic target. Given that CDK11 is expressed in various organs [14], potential side effects are a concern. However, no overt clinical signs of toxicity, including body weight loss, were observed during the treatment period, and all mice tolerated the treatment. Importantly, OTS964 demonstrated significant therapeutic efficacy as a single-agent treatment in this RTK model.

### 3.3. OTS964 Induces Apoptosis and Cell Cycle Arrest Through p53 Upregulation and SF3B1-Mediated Splicing Inhibition in RTK Cell Lines

**Figure 2:**
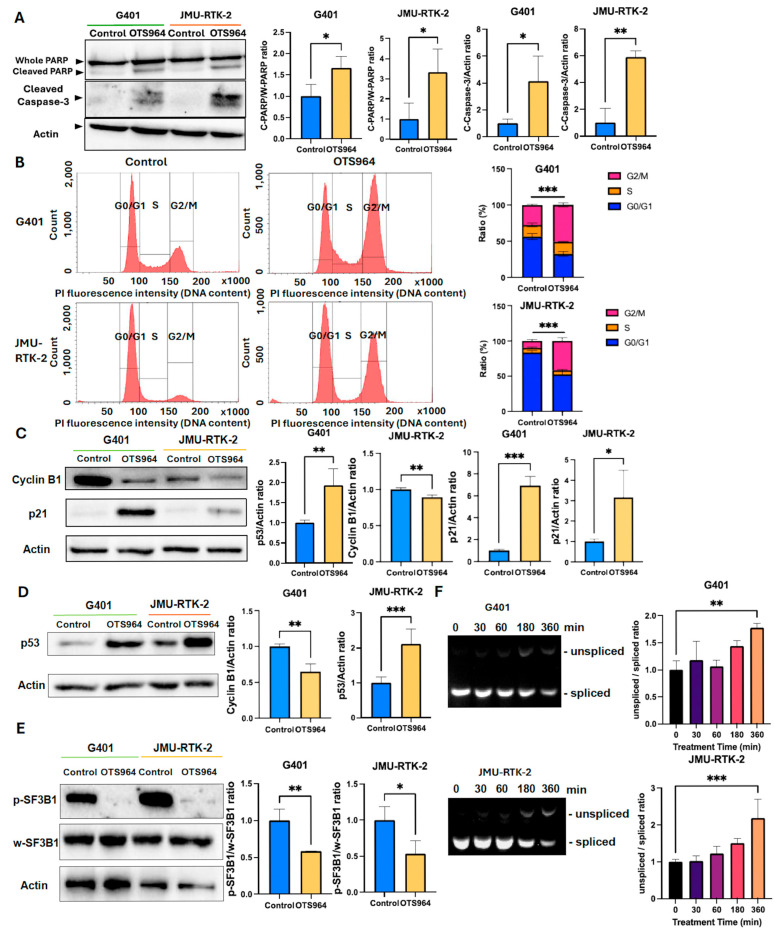
Mechanism analysis of OTS964 in RTK cell lines. (**A**) Immunoblot analysis of cleaved PARP (n = 3) and cleaved caspase-3 (n = 3) at 24 h after OTS964 treatment. (**B**) Flow cytometry analysis with double-thymidine block at 8 and 12 h after OTS964 treatment in G401 and JMU-RTK-2 cells, respectively (n = 3). Left panels show representative DNA content histograms plotted as event counts, and right panels show quantitative bar graphs representing the percentage of cells in G0/G1, S, and G2/M phases, normalized to total acquired events. A total of 50,000 events were collected for each sample. (**C**) Immunoblot analysis of cyclin B1 (n = 3) and p21 (n = 3) at 24 h after OTS964 treatment. (**D**) Immunoblot analysis of p53 in both cell lines at 24 h after OTS964 treatment (n = 5). (**E**) Immunoblot analysis of SF3B1 phosphorylation in G401 and JMU-RTK-2 cells at 24 h after OTS964 treatment (n = 3). (**F**) RT-PCR analysis of spliced and unspliced *DNAJB1* transcripts after OTS964 treatment (n = 3). Cells were treated with 80 nM OTS964 for G401 and 30 nM for JMU-RTK-2. Data are presented as the mean ± SD. * *p* < 0.05, ** *p* < 0.01, *** *p* < 0.001. The uncropped blots are shown in Figures S2–S5.

OTS964 treatment, at approximately IC80 concentrations; 80 nM for G401 and 30 nM for JMU-RTK-2, significantly increased the expression of cleaved PARP and cleaved caspase-3 (Figure 2A). It also induced G2/M cell cycle arrest, as demonstrated by a cell cycle assay with a double-thymidine block in both cell lines (Figure 2B). Consistent with G2/M arrest, OTS964 markedly decreased cyclin B1 expression and increased p21 expression (Figure 2C). Additionally, these effects were accompanied by increased expression of p53, a key regulator of apoptosis and cell cycle and an upstream regulator of p21 (Figure 2D).

OTS964 also significantly suppressed the phosphorylation of SF3B1, a core component of the spliceosome (Figure 2E). SF3B1 inhibition by OTS964 resulted in impaired RNA splicing efficiency, confirmed by increased accumulation of the unspliced transcript of *DNAJB1*, a known SF3B1-dependent transcript (Figure 2F) [25,27,28]. These findings demonstrate that OTS964 exerts its anti-tumor effects in RTK cells through multiple mechanisms: induction of apoptosis via caspase-3 activation, G2/M cell cycle arrest, p53 upregulation, and disruption of RNA splicing through SF3B1 inhibition.

## 4. Discussion

We identified CDK11 as a therapeutic target in RTK, based on the overexpression and prognostic significance of *CDK11B*. OTS964 exhibited potent anti-tumor activity, suppressing RTK cell growth in vitro and prolonging survival in xenograft models without marked toxicities. This is the first demonstration of therapeutic efficacy of CDK11 inhibition against RTK, supporting its clinical development potential. Mechanistically, OTS964 induces cell cycle arrest and disrupts RNA splicing via the SF3B1 pathway. These findings are consistent with the established biological effects of CDK11 inhibition, including disruption of RNA splicing, G2/M cell cycle arrest, and induction of apoptosis [10,11,12,13,14]. Although each mechanism has been reported separately in different cancers, our study is the first to show their concurrent occurrence, both leading RTK cells to apoptosis. The dual targeting of cell cycle progression and RNA splicing reflects the central roles of CDK11 in both processes, making it particularly well-suited for treating RTK, where both pathways are dysregulated due to *SMARCB1* loss [5,10].

The association between *CDK11B* expression and poor prognosis in RTK raises the question of how the two highly homologous paralogs, *CDK11A* and *CDK11B*, may be differentially regulated in cancer. Although they share nearly identical protein sequences, previous studies have shown that *CDK11A* and *CDK11B* are controlled by distinct promoter elements and transcription factors, suggesting context-dependent regulation at the transcriptional level [14]. Such differential regulation may underlie the observed upregulation of *CDK11B* in RTK. While the regulatory mechanisms remain to be fully elucidated, these findings provide a plausible biological basis for the differential prognostic relevance of *CDK11B* in RTK observed in this study.

OTS964 induced p53 upregulation in p53 wild-type RTK cell lines. Importantly, p53 mutation in RTK is rare, occurring in approximately 3% of rhabdoid tumors [29,30]. Previous studies have suggested that targeting the p53 pathway could be an effective therapeutic strategy in RTK [6,31,32,33]. Moreover, a recent study further demonstrated that CDK11 inhibition stabilizes and increases p53 proteins and induces aberrant p21 splicing through SF3B1 dephosphorylation, indicating that CDK11 regulates p53 signaling and RNA splicing in an integrated manner [33]. These results are consistent with our findings that OTS964 induces p53 upregulation and SF3B1 dephosphorylation in RTK cells. OTS964 induced multiple antitumor cellular responses in our model, including G2/M cell cycle arrest and disruption of RNA splicing via SF3B1 dephosphorylation. Although the precise functional relationship between these responses remains to be determined, these results suggest that p53 upregulation could represent one component of the cellular response to CDK11 inhibition, acting alongside cell cycle arrest and splicing dysregulation to mediate antitumor effects in RTK.

Genetic depletion of *CDK11B* in RTK cells could further strengthen causal inference. Prior genetic studies have firmly established CDK11 as the bona fide target of OTS964, demonstrating that *CDK11* depletion recapitulates OTS964 treatment and that OTS964 activity is abolished in *CDK11*-deficient cells in the A375 melanoma cell line [18]. Moreover, our demonstration of SF3B1 dephosphorylation and splicing disruption provides mechanistic evidence of on-target CDK11 inhibition in RTK. We also used *DNAJB1* intron retention as a surrogate marker of splicing inhibition as it serves as an established molecular marker of global SF3B1-dependent splicing disruption [27,28]. Importantly, apoptosis induced by CDK11 inhibition is likely mediated by the convergence of multiple cellular responses, including global splicing dysregulation and cell cycle arrest, rather than by a single, specific apoptosis-driving splicing event. Accordingly, identification of specific apoptosis-driving splicing events in RTK represents an important direction for future studies.

Notably, OTS964, as a single-agent treatment, and at doses lower than those reported in other cancer models [19,26,34], achieved significant tumor growth suppression and survival prolongation, suggesting that RTK may be particularly sensitive to CDK11 inhibition. The multi-target effects, including cell cycle arrest, RNA splicing inhibition, and p53 upregulation, likely contribute to the marked anti-tumor activity observed in our RTK model. At the tested dose and schedule, OTS964 was tolerated without overt clinical toxicity based on body weight and general condition monitoring. Detailed pharmacokinetic analyses and hematological or organ-specific toxicity assessments will be essential for further dose optimization and for advancing CDK11 inhibition toward translational development. The observed survival benefit demonstrates meaningful in vivo biological activity and supports CDK11 as a therapeutic target in RTK. While single-agent therapy alone is rarely sufficient for durable cancer control, these findings provide a strong foundation for future therapeutic optimization. Prior studies have shown that optimized drug delivery strategies, such as liposomal or nanoparticle-based formulations, can improve tolerability and enable more effective dosing of cytotoxic agents, including OTS964 [35,36,37]. In addition, rational combination strategies may further enhance therapeutic effects and potentially allow for a dose reduction in standard cytotoxic agents, thereby improving tolerability. Together, these approaches represent logical directions for future work to build on the significant single-agent efficacy observed in this study.

Given the shared molecular features of RTK and other rhabdoid tumors, CDK11 inhibition may have therapeutic potential in other RT subtypes. Together, our findings support CDK11 as a promising therapeutic target in RTK and provide a rationale for future translational and clinical studies.

## 5. Conclusions

This study identified CDK11 as a promising therapeutic target in RTK, where CDK11B is overexpressed and associated with poor prognosis. The CDK11 inhibitor OTS964, as a single drug therapy, demonstrated potent anti-tumor efficacy in vitro and in vivo, with minimal toxicity. Mechanistically, OTS964 induces G2/M cell cycle arrest, p53 upregulation, and the inhibition of RNA splicing via SF3B1 dephosphorylation and promotes apoptosis. Given these findings, CDK11 inhibition represents a promising therapeutic strategy for RTK and other *SMARCB1*-deficient rhabdoid tumors. Future studies should focus on optimizing drug delivery and exploring combination strategies to enhance the clinical potential of CDK11 inhibition.

## Data Availability

Data are contained within the article and its Supplementary Materials.

## Supplementary Materials

The following supporting information can be downloaded at: https://www.mdpi.com/article/10.3390/cancers18020261/s1, Figure S1: CDK11B expression in RTK, WT, and CCSK and overall survival of WT and CCSK patients stratified by *CDK11B* expression; Figure S2: Uncropped Western blots corresponding to Figure 2A; Figure S3: Uncropped Western blots corresponding to Figure 2C; Figure S4: Uncropped Western blots corresponding to Figure 2D,E; Figure S5: Uncropped RT-PCR blots corresponding to Figure 2F; File S1: Raw data used for statistical analyses in this study.

## Abbreviations

The following abbreviations are used in this manuscript:

RTK	Rhabdoid tumor of the kidney
RT	Rhabdoid tumor
AT/RT	Atypical teratoid/Rhabdoid tumor
MRT	Malignant rhabdoid tumor
JCRB	Japanese Collection of Research Bioresources
SRB	Sulforhodamine B
IACUC	Institutional Animal Care and Use Committee
H&E	Hematoxylin and Eosin
RT-PCR	Reverse-Transcription-PCR
TARGET	Therapeutically Applicable Research To Generate Effective Treatments
WT	Wilms tumor
CCSK	Clear cell sarcoma of the kidney

## References

[1] Geller J.I., Roth J.J., Biegel J.A. (2015). Biology and Treatment of Rhabdoid Tumor. Crit. Rev. Oncog..

[2] Heck J.E., Lombardi C.A., Cockburn M., Meyers T.J., Wilhelm M., Ritz B. (2013). Epidemiology of rhabdoid tumors of early childhood: Rhabdoid Tumors of Early Childhood. Pediatr. Blood Cancer.

[3] Nemes K., Johann P.D., Tüchert S., Melchior P., Vokuhl C., Siebert R., Furtwängler R., Frühwald M.C. (2022). Current and emerging therapeutic approaches for extracranial malignant rhabdoid tumors. Cancer Manag. Res..

[4] Frühwald M.C., Biegel J.A., Bourdeaut F., Roberts C.W., Chi S.N. (2016). Atypical teratoid/rhabdoid tumors—Current concepts, advances in biology, and potential future therapies. Neuro Oncol..

[5] Liu N.Q., Paassen I., Custers L., Zeller P., Teunissen H., Ayyildiz D., He J., Buhl J.L., Hoving E.W., Van Oudenaarden A. (2023). SMARCB1 loss activates patient-specific distal oncogenic enhancers in malignant rhabdoid tumors. Nat. Commun..

[6] Carugo A., Minelli R., Sapio L., Soeung M., Carbone F., Robinson F.S., Tepper J., Chen Z., Lovisa S., Svelto M. (2019). p53 Is a Master Regulator of Proteostasis in SMARCB1-Deficient Malignant Rhabdoid Tumors. Cancer Cell.

[7] Kim K.H., Roberts C.W.M. (2014). Mechanisms by which SMARCB1 loss drives rhabdoid tumor growth. Cancer Genet..

[8] Hsu T.Y.T., Simon L.M., Neill N.J., Marcotte R., Sayad A., Bland C.S., Echeverria G.V., Sun T., Kurley S.J., Tyagi S. (2015). The spliceosome is a therapeutic vulnerability in MYC-driven cancer. Nature.

[9] Iwai K., Yaguchi M., Nishimura K., Yamamoto Y., Tamura T., Nakata D., Dairiki R., Kawakita Y., Mizojiri R., Ito Y. (2018). Anti-tumor efficacy of a novel CLK inhibitor via targeting RNA splicing and MYC-dependent vulnerability. EMBO Mol. Med..

[10] Blazek D. (2023). Therapeutic potential of CDK11 in cancer. Clin. Transl. Med..

[11] Loyer P., Trembley J.H. (2020). Roles of CDK/Cyclin complexes in transcription and pre-mRNA splicing: Cyclins L and CDK11 at the cross-roads of cell cycle and regulation of gene expression. Semin. Cell Dev. Biol..

[12] Hluchý M., Gajdušková P., Ruiz De Los Mozos I., Rájecký M., Kluge M., Berger B.T., Slabá Z., Potěšil D., Weiß E., Ule J. (2022). CDK11 regulates pre-mRNA splicing by phosphorylation of SF3B1. Nature.

[13] Hluchý M., Blazek D. (2024). CDK11, a splicing-associated kinase regulating gene expression. Trends Cell Biol..

[14] Zhou Y., Shen J.K., Hornicek F.J., Kan Q., Duan Z. (2016). The emerging roles and therapeutic potential of cyclin-dependent kinase 11 (CDK11) in human cancer. Oncotarget.

[15] Liu X., Gao Y., Shen J., Yang W., Choy E., Mankin H., Hornicek F.J., Duan Z. (2016). Cyclin-Dependent Kinase 11 (CDK11) Is Required for Ovarian Cancer Cell Growth In Vitro and In Vivo, and Its Inhibition Causes Apoptosis and Sensitizes Cells to Paclitaxel. Mol. Cancer Ther..

[16] Du Y., Yan D., Yuan Y., Xu J., Wang S., Yang Z., Cheng W., Tian X., Kan Q. (2019). CDK11p110 plays a critical role in the tumorigenicity of esophageal squamous cell carcinoma cells and is a potential drug target. Cell Cycle.

[17] Kren B.T., Unger G.M., Abedin M.J., Vogel R.I., Henzler C.M., Ahmed K., Trembley J.H. (2015). Preclinical evaluation of cyclin dependent kinase 11 and casein kinase 2 survival kinases as RNA interference targets for triple negative breast cancer therapy. Breast Cancer Res..

[18] Kelso S., O’Brien S., Kurinov I., Angers S., Sicheri F. (2022). Crystal structure of the CDK11 kinase domain bound to the small-molecule inhibitor OTS964. Structure.

[19] Matsuo Y., Park J.H., Miyamoto T., Yamamoto S., Hisada S., Alachkar H., Nakamura Y. (2014). TOPK inhibitor induces complete tumor regression in xenograft models of human cancer through inhibition of cytokinesis. Sci. Transl. Med..

[20] Lin A., Giuliano C.J., Palladino A., John K.M., Abramson C., Yuan M.L., Sausville E.L., Lukow D.A., Liu L., Chait A.R. (2019). Off-target toxicity is a common mechanism of action of cancer drugs undergoing clinical trials. Sci. Transl. Med..

[21] Vichai V., Kirtikara K. (2006). Sulforhodamine B colorimetric assay for cytotoxicity screening. Nat. Protoc..

[22] Lee A.Q., Konishi H., Ijiri M., Li Y., Panigrahi A., Chien J., Satake N. (2024). Therapeutic efficacy of RAS inhibitor trametinib using a juvenile myelomonocytic leukemia patient-derived xenograft model. Pediatr. Hematol. Oncol..

[23] Goldman M.J., Craft B., Hastie M., Repečka K., McDade F., Kamath A., Banerjee A., Luo Y., Rogers D., Brooks A.N. (2020). Visualizing and interpreting cancer genomics data via the UCSC Xena platform. Nat. Biotechnol..

[24] Yanagisawa S., Kadouchi I., Yokomori K., Hirose M., Hakozaki M., Hojo H., Maeda K., Kobayashi E., Murakami T. (2009). Identification and Metastatic Potential of Tumor-Initiating Cells in Malignant Rhabdoid Tumor of the Kidney. Clin. Cancer Res..

[25] Larrayoz M., Blakemore S.J., Dobson R.C., Blunt M.D., Rose-Zerilli M.J.J., Walewska R., Duncombe A., Oscier D., Koide K., Forconi F. (2016). The SF3B1 inhibitor spliceostatin A (SSA) elicits apoptosis in chronic lymphocytic leukaemia cells through downregulation of Mcl-1. Leukemia.

[26] Choi H., Cao J., Qiao H., Chen I., Zhou R. (2022). Improving Cancer Detection and Treatment by pH-Sensitive Peptide Nanoparticle Drug Delivery Platform: Pharmacokinetics, Toxicity, and Immunogenicity Profile. Adv. NanoBiomed Res..

[27] Kotake Y., Sagane K., Owa T., Mimori-Kiyosue Y., Shimizu H., Uesugi M., Ishihama Y., Iwata M., Mizui Y. (2007). Splicing factor SF3b as a target of the antitumor natural product pladienolide. Nat. Chem. Biol..

[28] Kashyap M.K., Kumar D., Villa R., La Clair J.J., Benner C., Sasik R., Jones H., Ghia E.M., Rassenti L.Z., Kipps T.J. (2015). Targeting the spliceosome in chronic lymphocytic leukemia with the macrolides FD-895 and pladienolide-B. Haematologica.

[29] Ng J.M.Y., Martinez D., Marsh E.D., Zhang Z., Rappaport E., Santi M., Curran T. (2015). Generation of a Mouse Model of Atypical Teratoid/Rhabdoid Tumor of the Central Nervous System through Combined Deletion of Snf5 and p53. Cancer Res..

[30] Lee R.S., Stewart C., Carter S.L., Ambrogio L., Cibulskis K., Sougnez C., Lawrence M.S., Auclair D., Mora J., Golub T.R. (2012). A remarkably simple genome underlies highly malignant pediatric rhabdoid cancers. J. Clin. Investig..

[31] Cooper G.W., Hong A.L. (2022). SMARCB1-Deficient Cancers: Novel Molecular Insights and Therapeutic Vulnerabilities. Cancers.

[32] Howard T.P., Arnoff T.E., Song M.R., Giacomelli A.O., Wang X., Hong A.L., Dharia N.V., Wang S., Vazquez F., Pham M.T. (2019). MDM2 and MDM4 Are Therapeutic Vulnerabilities in Malignant Rhabdoid Tumors. Cancer Res..

[33] Alimova I., Wang D., Danis E., Pierce A., Donson A., Serkova N., Madhavan K., Lakshmanachetty S., Balakrishnan I., Foreman N. (2022). Targeting the TP53/MDM2 axis enhances radiation sensitivity in atypical teratoid rhabdoid tumors. Int. J. Oncol..

[34] Krejcir R., Arcimowicz L., Martinkova L., Hrabal V., Zavadil Kokas F., Henek T., Kucerikova M., Bonczek O., Zatloukalova P., Hernychova L. (2025). CDK 11 inhibition induces cytoplasmic p21 WAF 1 splice variant by p53 stabilisation and SF 3 B 1 inactivation. Mol. Oncol..

[35] Pirovano G., Roberts S., Brand C., Donabedian P.L., Mason C., de Souza P.D., Higgins G.S., Reiner T. (2019). [18F]FE-OTS964: A Small Molecule Targeting TOPK for In Vivo PET Imaging in a Glioblastoma Xenograft Model. Mol. Imaging Biol..

[36] Federman N., Denny C.T. (2010). Targeting Liposomes Toward Novel Pediatric Anticancer Therapeutics. Pediatr. Res..

[37] Gilabert-Oriol R., Sutherland B.W., Anantha M., Pallaoro A., Bally M.B. (2019). Liposomal OTS964, a TOPK inhibitor: A simple method to estimate OTS964 association with liposomes that relies on enhanced OTS964 fluorescence when bound to albumin. Drug Deliv. Transl. Res..

