# Supplementary material for: Targeting CDK11 in Rhabdoid Tumor of the Kidney
Source: Cancers (Basel). 2026 Jan 14;18(2):261. doi: 10.3390/cancers18020261 (PMC12839316; doi:10.3390/cancers18020261)
Supplement: Supplementary file 1 [file cancers-18-00261-s001.zip › Supplementary_Figures.pptx]

## Slide 1
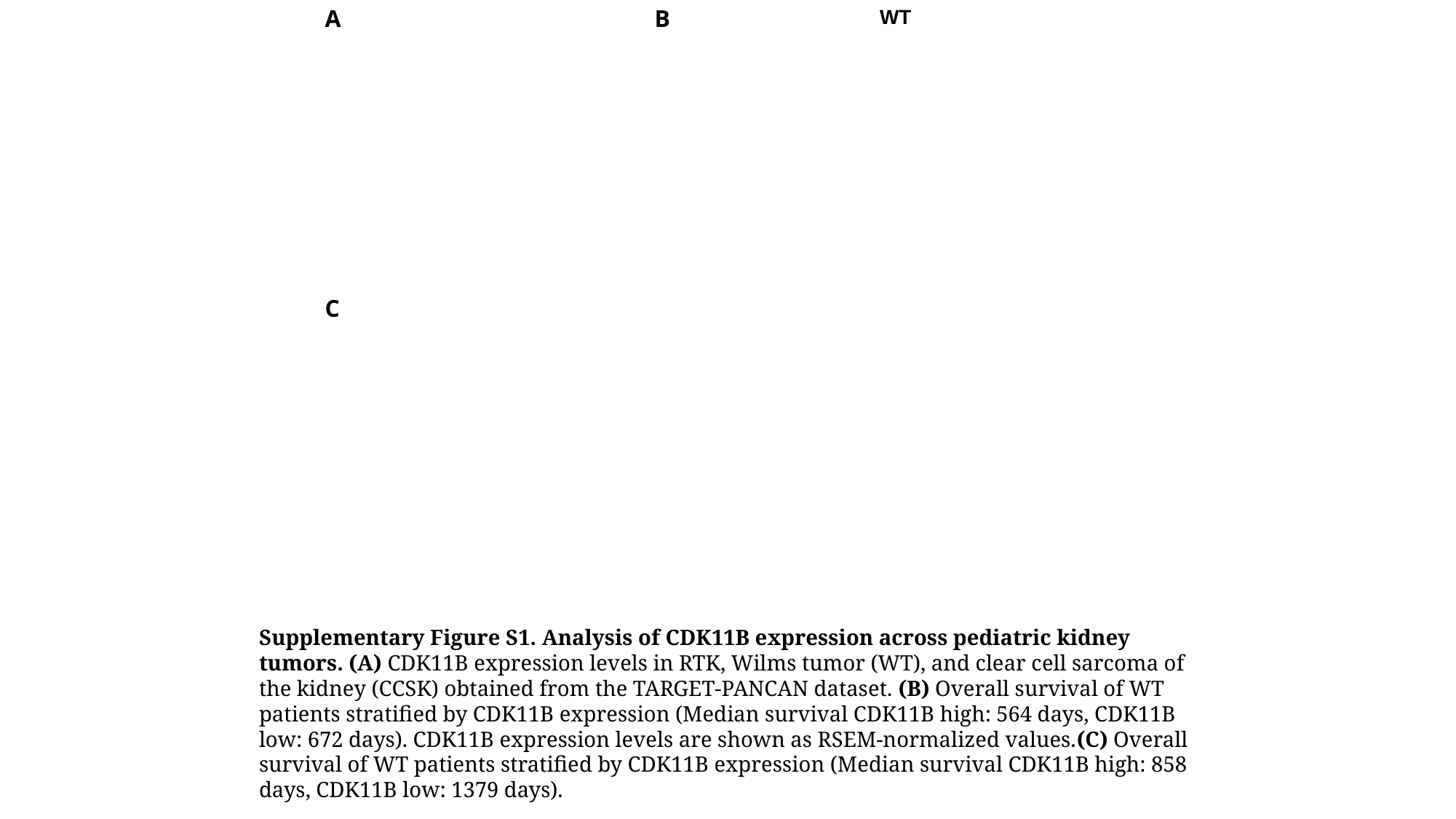

B
A
WT
C
Supplementary Figure S1. Analysis of CDK11B expression across pediatric kidney tumors. (A) CDK11B expression levels in RTK, Wilms tumor (WT), and clear cell sarcoma of the kidney (CCSK) obtained from the TARGET-PANCAN dataset. (B) Overall survival of WT patients stratified by CDK11B expression (Median survival CDK11B high: 564 days, CDK11B low: 672 days). CDK11B expression levels are shown as RSEM-normalized values.(C) Overall survival of WT patients stratified by CDK11B expression (Median survival CDK11B high: 858 days, CDK11B low: 1379 days).
100x
400x

## Slide 2
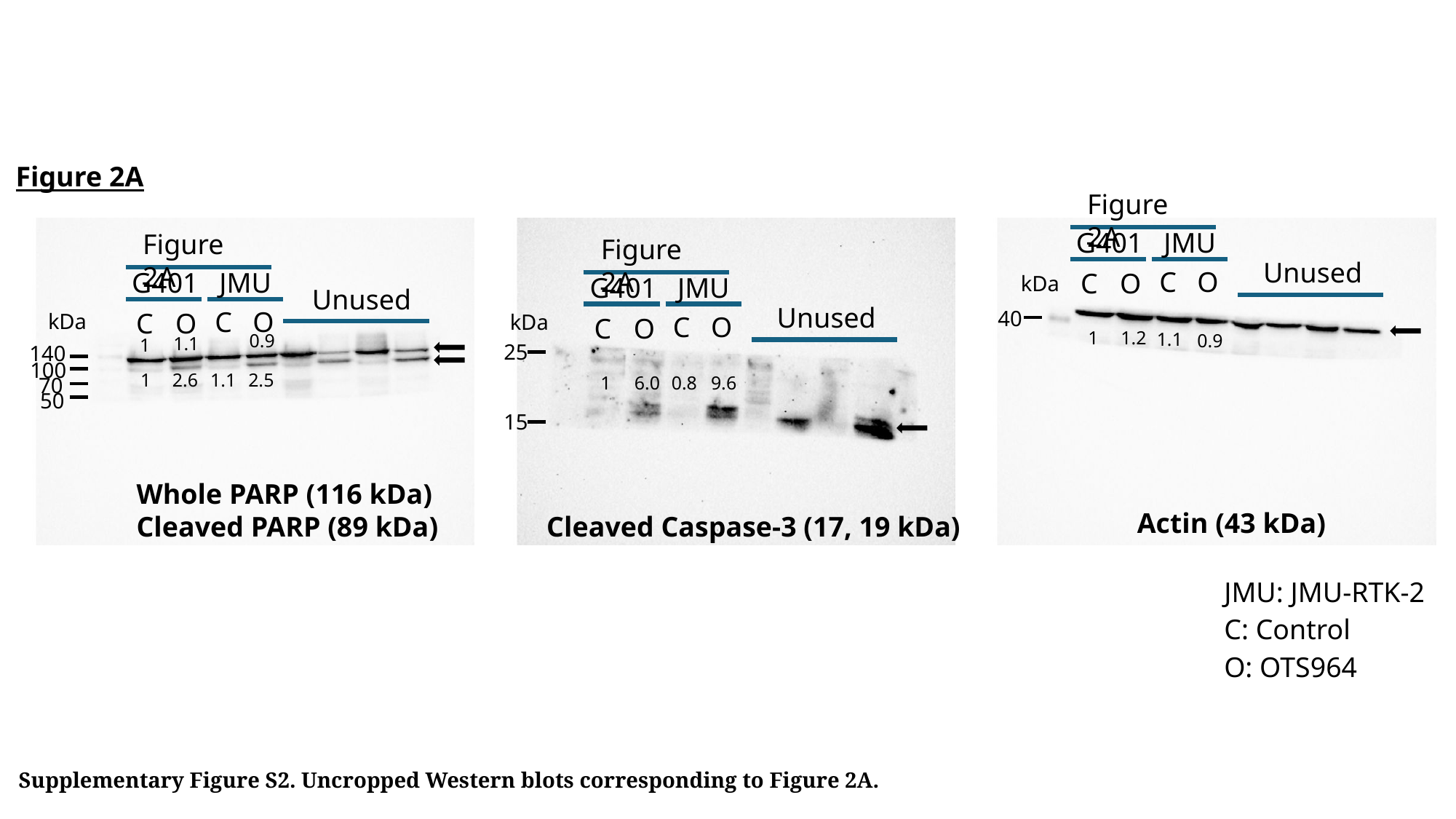

Figure 2A
Figure 2A
G401
JMU
Figure 2A
Figure 2A
Unused
C
O
G401
JMU
C
O
G401
JMU
kDa
Unused
Unused
C
O
40
C
O
kDa
kDa
C
O
C
O
1
1.2
1.1
0.9
0.9
1.1
1
25
140
100
1
2.6
1.1
2.5
1
6.0
0.8
9.6
70
50
15
Whole PARP (116 kDa)
Cleaved PARP (89 kDa)
Actin (43 kDa)
Cleaved Caspase-3 (17, 19 kDa)
JMU: JMU-RTK-2
C: Control
O: OTS964
Supplementary Figure S2. Uncropped Western blots corresponding to Figure 2A.

## Slide 3
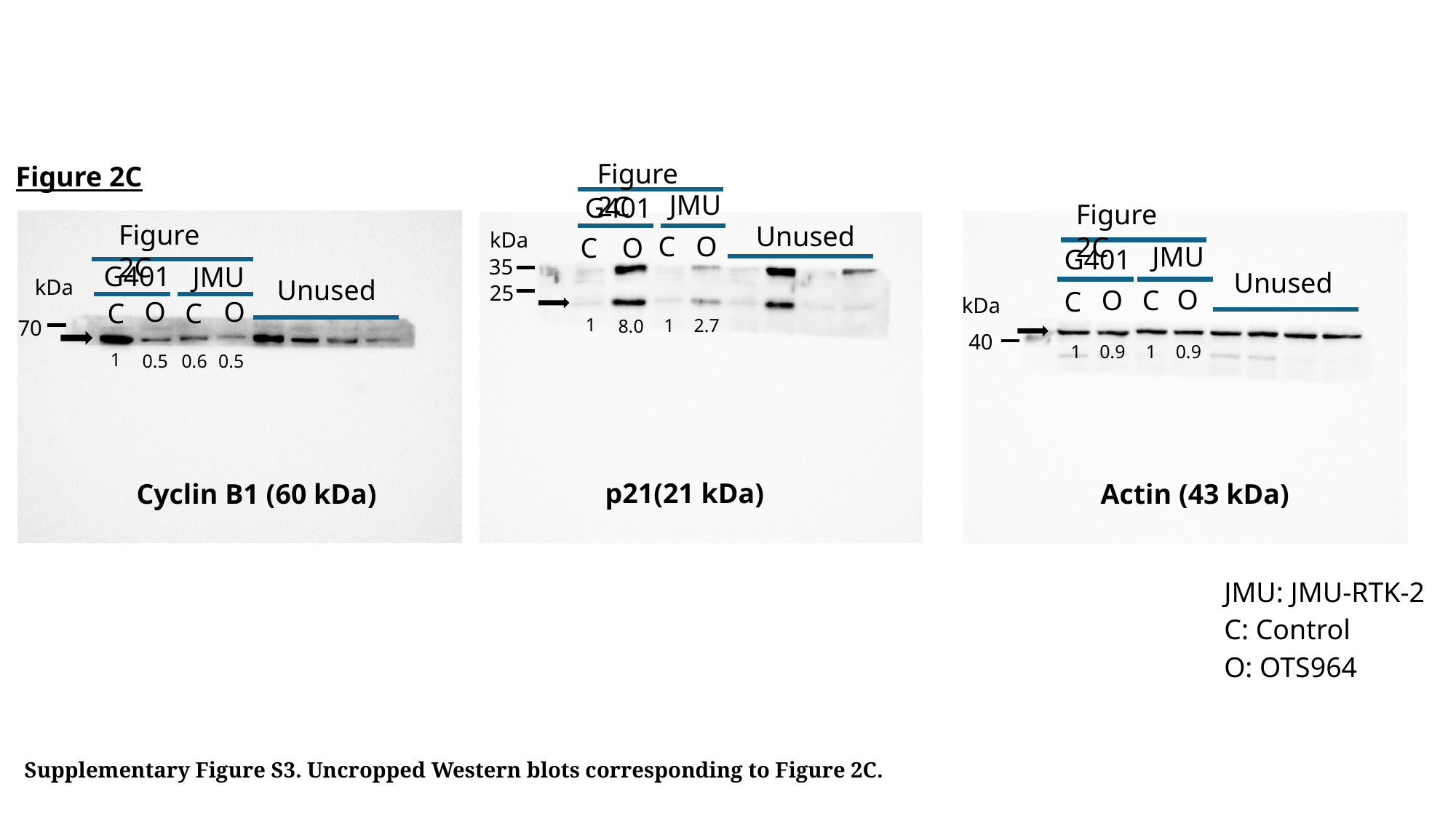

Figure 2C
Figure 2C
JMU
G401
Figure 2C
Figure 2C
Unused
kDa
C
O
C
O
JMU
G401
35
G401
JMU
Unused
Unused
kDa
25
O
C
O
C
kDa
O
O
C
C
1
1
2.7
70
8.0
40
0.9
1
0.9
1
1
0.5
0.6
0.5
p21(21 kDa)
Cyclin B1 (60 kDa)
Actin (43 kDa)
JMU: JMU-RTK-2
C: Control
O: OTS964
Supplementary Figure S3. Uncropped Western blots corresponding to Figure 2C.

## Slide 4
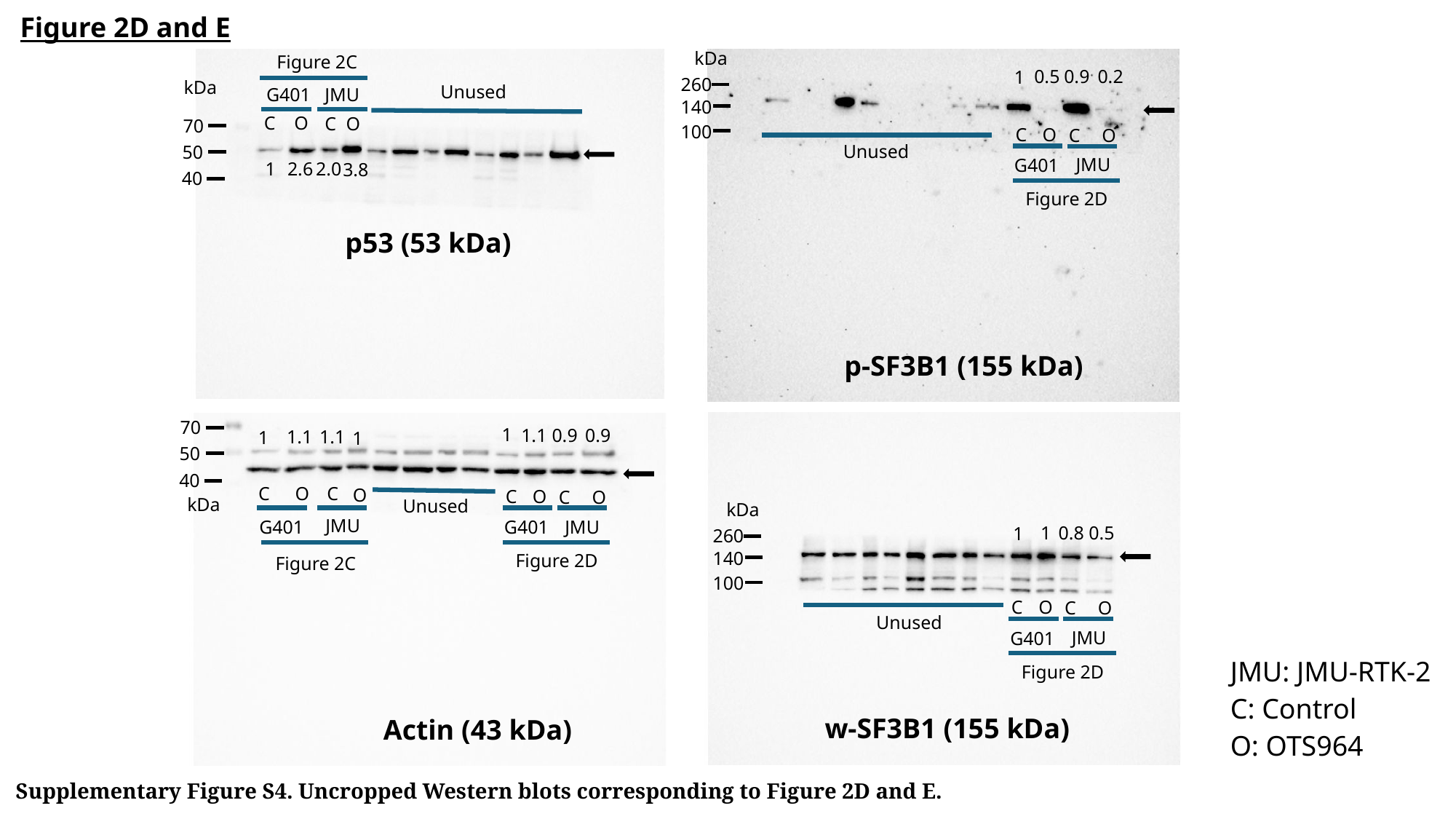

Figure 2D and E
kDa
Figure 2C
0.5
0.9
0.2
1
260
kDa
Unused
G401
JMU
140
C
O
C
O
70
100
C
O
C
O
Unused
50
JMU
G401
1
2.6
2.0
3.8
40
Figure 2D
p53 (53 kDa)
p-SF3B1 (155 kDa)
70
1
0.9
0.9
1.1
1.1
1.1
1
1
50
40
C
O
C
O
C
O
C
O
kDa
Unused
kDa
JMU
JMU
G401
G401
1
0.8
0.5
1
260
140
Figure 2D
Figure 2C
100
C
O
C
O
Unused
JMU
G401
JMU: JMU-RTK-2
Figure 2D
C: Control
w-SF3B1 (155 kDa)
Actin (43 kDa)
O: OTS964
Supplementary Figure S4. Uncropped Western blots corresponding to Figure 2D and E.

## Slide 5
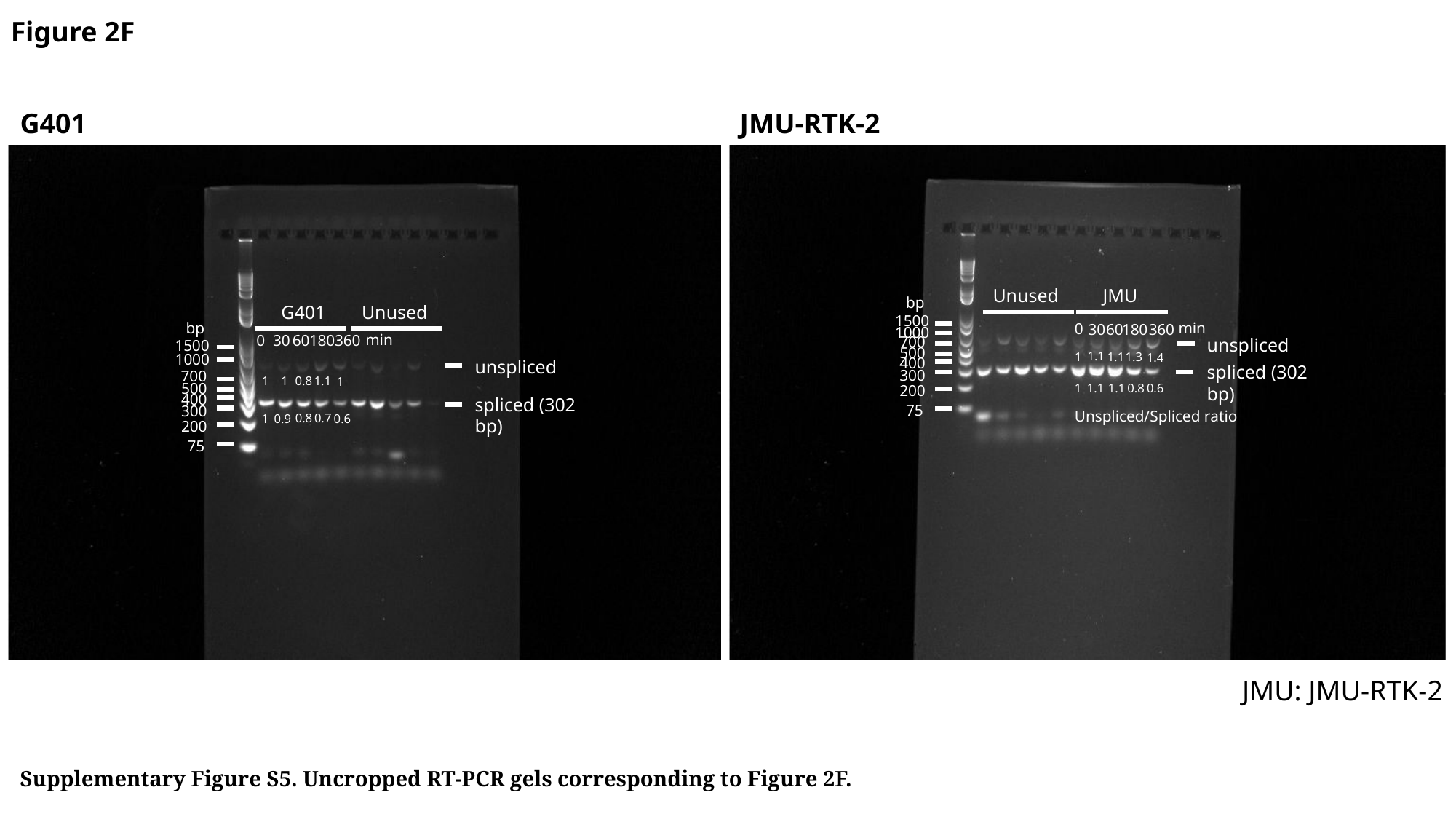

Figure 2F
G401
JMU-RTK-2
Unused
JMU
bp
G401
Unused
1500
bp
min
0
60
180
360
30
1000
min
0
30
60
360
180
700
unspliced
1500
500
1.1
1
1.1
1.3
1.4
1000
400
unspliced
spliced (302 bp)
300
700
1
1
0.8
1.1
1
500
1.1
0.8
200
1
1.1
0.6
400
spliced (302 bp)
75
300
Unspliced/Spliced ratio
0.8
0.7
1
0.9
0.6
200
75
JMU: JMU-RTK-2
Supplementary Figure S5. Uncropped RT-PCR gels corresponding to Figure 2F.
